# Evaluating the performance of the Narcotrend^R^ EEG index during anaesthesia for cardiothoracic surgery: a single-centre retrospective study

**DOI:** 10.1186/s12871-025-03500-5

**Published:** 2025-11-14

**Authors:** Max Ebensperger, Matthias Kreuzer, Stephan Kratzer, Darren Hight, Heiko A. Kaiser, Gerhard Schneider, Stefan Schwerin

**Affiliations:** 1 Department of Anesthesiology and Intensive Care, TUM School of Medicine and Health, Technical University of Munich, Munich, Germany; 2 Department of Anesthesia, Intensive Care and Pain Medicine, Hessing Foundation, Augsburg, Germany; 3 Department of Anesthesiology and Pain Medicine, Inselspital, Bern University Hospital, University of Bern, Bern, Switzerland; 4 Center for Anaesthesiology and Intensive Care Medicine, Hirslanden Clinic Aarau, Aarau, Switzerland

**Keywords:** Anaesthesia, Cardiac surgery, Neuromonitoring, Electroencephalogram, Narcotrend, Burst suppression

## Abstract

**Background:**

Using neuromonitoring during general anaesthesia provides insights into the effects of anaesthetics on the brain. We focus on the performance of the processed EEG indices Narcotrend^R^ (NCT) and Burst Suppression Ratio (BSR) of the Narcotrend-Compact-M^R^ Module, which serve as surrogate parameters for the level of consciousness.

**Methods:**

In this single-centre retrospective study, we analysed processed electroencephalographic (EEG) data from 439 patients who underwent general anaesthesia for cardiac surgery. We employed data visualisation techniques, such as histograms and heat maps. We conducted statistical analyses using correlation coefficients, receiver operating characteristics, and linear regression to evaluate Narcotrend performance under various BSR conditions.

**Results:**

The NCT index demonstrated distinct “*peak*” values (37, 46, and 61), which occurred with a probability more than two standard deviations above the overall index distribution (BSR = 0). During steady-state anaesthesia, 70% [Q1, Q3: 67,72] of values were within the manufacturer-recommended range for adequate anaesthesia, 22% [Q1, Q3: 21,29] were below, and 8% [Q1, Q3: 6,12] were above. With the onset of BSR > 0, NCT decreased significantly (*p* < 0.001) but showed significant variability immediately before and after automated burst suppression detection. Approximately 13% [Q1, Q3: 9,24] of NCT readings were non-interpretable. These brief episodes increased significantly with patient age (*p* = 0.013) and were not attributable to concurrent burst suppression.

**Conclusion:**

The Narcotrend index remains within recommended ranges during steady-state anaesthesia in a predominantly male patient cohort undergoing cardiac surgery. However, index performance decreases with age, and the high incidence of non-interpretable readings in elderly patients highlights the need for cautious interpretation, despite their short duration. Automatically detected burst suppression (BSR > 0) leads to a near-instant decrease in NCT values, suggesting a technical link between the algorithms. “*Peak*” index values indicate an irregular scaling in the distribution of NCT index values.

**Trial registration:**

This trial was retrospectively registered at ClinicalTrials.gov (NCT02976584) in October 2016.

**Supplementary Information:**

The online version contains supplementary material available at 10.1186/s12871-025-03500-5.

## Introduction

### Background

Processed electroencephalogram (EEG) indices are intended to assist anaesthesiologists in managing anaesthesia and are endorsed by international guidelines [1], albeit with varying strengths of recommendation. It is assumed that the anaesthetic “*depth*” can be titrated according to clinical requirements. Processed indices aim to translate these changes into a continuous and dimensionless scale, facilitating interpretation and application in clinical settings [2]. This principle extends to the Narcotrend index (NCT), produced by the Narcotrend-Compact M^R^ Module (Narcotrend-Gruppe, Hannover, Germany). Raw EEG signals are recorded from a patient’s forehead and filtered to within the 0.5–45 Hz range. The classification (stages F–A and 0–100) is based on trace segments lasting 20 s each with a 75% overlap [3]. Respective thresholds were validated visually and via spectral and power analyses for volatile and intravenous anaesthetics [4]. Like other manufacturers, the Narcotrend defines a range of index values (NCT 37–64) considered indicative of adequate general anaesthesia, hereinafter referred to as “*adequate anaesthesia*” range. A debate revolves around whether using processed EEG indices for guiding anaesthesia can reduce intraoperative awareness [5] or postoperative delirium [6]. Specific EEG patterns, including burst suppression during maintenance, are associated with an increased risk for the latter [7]. The NCT is supplemented with the automated burst suppression ratio (BSR) to help signal overly “*deep*” anaesthetic levels. We evaluated the performance of processed, EEG-based indices from the Narcotrend monitor, which operates independently of anaesthetic regimens, in patients undergoing cardiac surgery, a setting known for complicating the reliability of processed indices [8]. These challenges stem from intraoperative hypothermia, the use of cardiopulmonary bypass (CPB), and complex procedures often associated with significant hemodynamic instability, major bleeding, and other critical complications. Additionally, cardiac surgeries are frequently performed on high-risk patients characterised by older age, frailty, comorbidities, or critical status, which are linked to elevated rates of neurocognitive dysfunction [9].

### Objectives

Our primary objective in this retrospective data analysis was to examine age-dependent and overall distribution patterns in NCT index values. We further assessed the conformity of recorded NCT values with the thresholds of *“adequate anaesthesia*”, as defined by the manufacturer. Additionally, we explored the performance of NCT in the presence of BSR > 0 and the frequency and distribution of non-interpretable NCT monitor outputs.

## Methods

### Patient population and index value processing

For this retrospective, single-centre study, we analysed processed intraoperative EEG data from 466 patients who underwent general anaesthesia for cardiac surgery with CPB. Of the study cohort, 24 patients declined international data transfer, and in 3 patients, NCT or BSR values were entirely absent. These cases were excluded from all subsequent analyses (see Supplementary Figure S1). We used subset data from the EPOCAS study conducted at the University Hospital of Bern, Switzerland [10–12]. Our dataset provided simultaneous EEG and NIRS recordings. NCT and BSR values were extracted from the Narcotrend M^R^ Module (Narcotrend-Gruppe, Hannover, Germany). Patient age was entered at the beginning of each case in the Narcotrend Monitor. To ease the interpretation of the BSR, which was recorded on the patient’s left and right forehead, we binned the respective values per patient and calculated a single mean BSR value. We used BSR≥5 as our primary threshold for clinically relevant burst suppression [13, 14], corresponding to approximately 3 s of suppression per analysed EEG segment. For selected analyses, we also included BSR>0; these instances are explicitly stated (see also Supplementary Figure S1). We present the BSR (left) and BSR (right) data in Supplementary Figure S2, S3 A, B. Our records included demographic information such as patient age, sex (male or female), Body Mass Index, and whether volatile anaesthetics or propofol were used for maintaining general anaesthesia. The trend data (NCT, BSR) were recorded at one-second intervals. The relationship between concentrations of anaesthetic agents and NCT values was reported previously [12]. Adjustments to anaesthetic dosing were made at the discretion of the attending anaesthesiologists, without predefined NCT or BSR titration targets.

### Analysed time period

Processed EEG monitoring and data collection were initiated only after anaesthesia induction and patient transfer to the operating room. Recordings were terminated at the end of surgery, while patients were still sedated and intubated for delayed extubation in the intensive care unit. Therefore, data were limited to the intraoperative period, and our analysis focused on steady-state anaesthesia. During CPB, anaesthesia was maintained using volatile anaesthetics only.

### Age bins and definition of “*peak*” index values

We grouped patients into aggregate age bins due to limited sample sizes. The first bin included ages 23–30, followed by 5-year increments until 80–85 (Supplementary Figure S4). To identify NCT values that showed a higher likelihood of occurrence, we calculated the mean probability of occurrence over the index range (0–100) and identified values that exceeded the mean by two standard deviations. We termed these NCT values as “*peak*” index values.

### Statistical analysis

Continuous data are presented as medians with first and third quartiles (Q1, Q3), while categorical data are shown as absolute numbers with percentages. We performed linear regression analyses using MATLAB’s “*fitlm*” function. Spearman’s rank correlation coefficients (rho) with corresponding 95^th^ percentile confidence intervals were determined using 10k bootstrapping iterations. The significance level was set at *p* < 0.05. We calculated the area under the curve (AUC) of the receiver operating characteristic (ROC) with 95% confidence intervals using the “*perfcurve*” function. A non-parametric Friedman test detected differences in mean NCT values across three conditions (immediately before BSR>0, during BSR>0, and immediately after BSR>0). Post-hoc pairwise comparisons were performed using the Wilcoxon signed-rank test. All analyses and visualisations were conducted using MATLAB R2023a (The MathWorks, Inc., Natick, MA, USA).

### Missing data and bias

We created value pairs by connecting simultaneously registered index values for NCT and BSR. Only numeric index values (0–100) were considered, while missing or non-interpretable entries were omitted. If a patient exhibited only non-interpretable outputs for one or both indices (NCT, BSR), the patient was excluded entirely (Supplementary Figure S1).

## Results

### Patient characteristics

The median age of our patient cohort was 67 [Q1,Q3: 58,73] years. 72% were male and 28% were female. The median surgery duration was 254 [Q1,Q3: 206,313] minutes. The median BMI was 26.8 [Q1,Q3: 23.7,30.8]. Histograms for surgery duration and age distributions are presented in Supplementary Figure S4. Anaesthesia was maintained with volatile anaesthetics for most cases: isoflurane 84%, isoflurane/sevoflurane 8.8%, sevoflurane 3.2%, isoflurane/desflurane 0.45%, and with propofol: 3.55%.

### Distribution of narcotrend index values

**Fig. 1:**
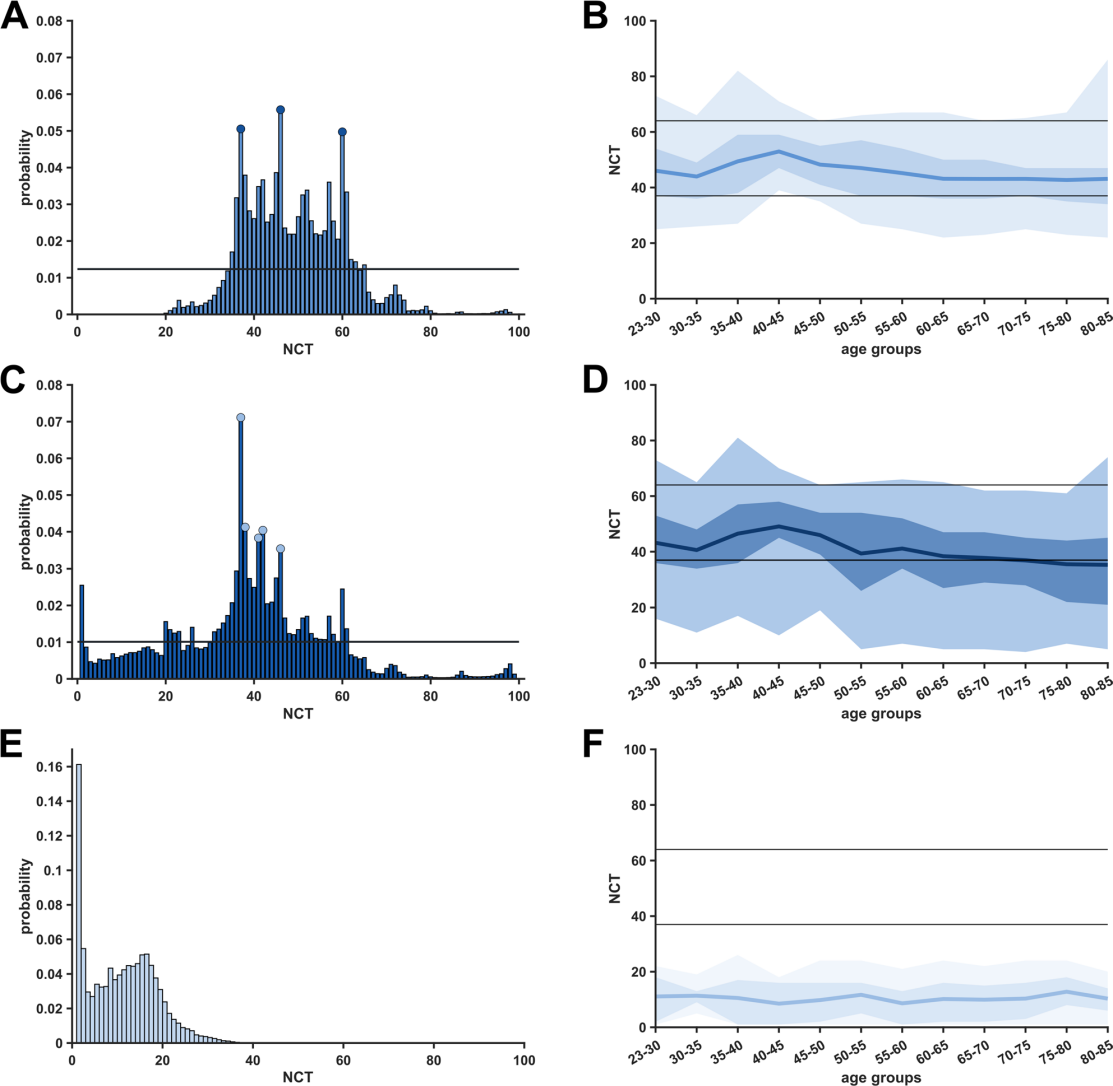
Histograms of Narcotrend (NCT) index value probabilities and mean NCT values over age groups. On the left, multiple index values show a higher probability than adjacent values, contrasting the expected continuous distribution. On the right, mean NCT values are plotted in dark colors, while the 75th and 95th percentiles of NCT are in corresponding lighter colors. The manufacturer-recommended index range (37–64) for “*adequate anaesthesia*” is marked with solid lines. **A** Histogram of probabilities for the NCT index range (BSR=0). “*Peak*” index values are marked with a circle (exceeding the mean probability by two standard deviations). **B** Mean NCT values (BSR=0) over age groups. **C** Histogram of probabilities for the NCT index range (BSR≥0). **D** Mean NCT values (BSR≥0) over age groups. **E** Histogram of probabilities for NCT index values (BSR≥5). **F** Mean NCT values (BSR≥5) over age groups

**Fig. 2:**
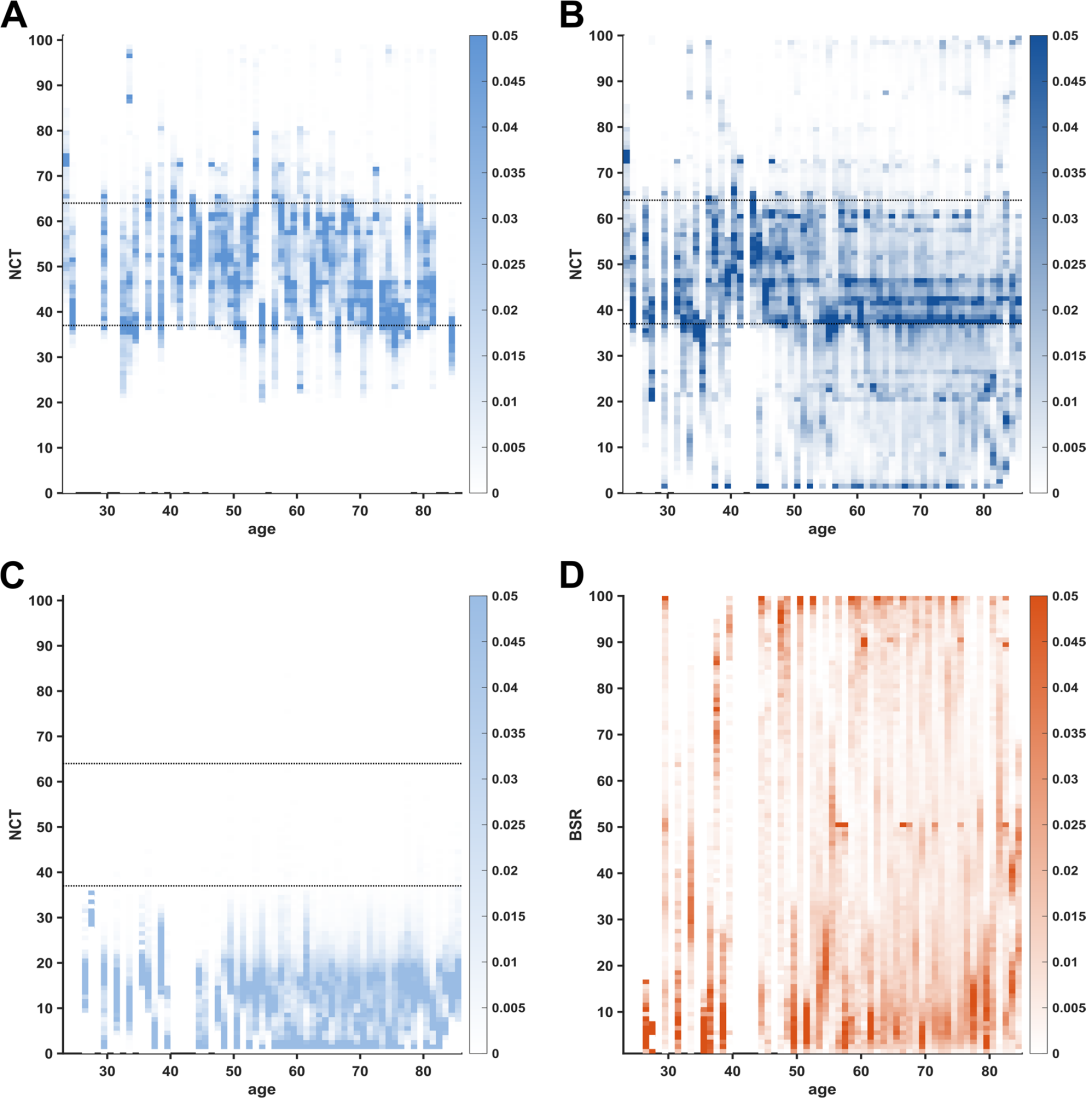
Heat maps of Narcotrend (NCT) and Burst Suppression Ratio (BSR) probability distributions as a function of age. A higher occurrence probability translates into darker shading. Missing age groups appear as blank vertical lines. The manufacturer-recommended range for “*adequate anaesthesia*” (37–64) is indicated with black dotted lines. Horizontal lines visually suggest “*peak*” index values. **A** Heat map of occurrence probability for NCT index values (BSR=0). **B** Heat map of occurrence probability for NCT index values (BSR≥0). **C** Heat map of occurrence probability for NCT index values (BSR≥5). **D** Heat map of occurrence probability for BSR index values

Our analysis of index value distributions revealed that specific NCT values were more likely to occur than adjacent values (which we termed “*peak*” index values). During steady-state anaesthesia (BSR=0), only NCT values 37, 46, and 61 showed a deviation greater than two standard deviations from the mean probability of index values (Fig. 1 A). When considering all NCT values (BSR ≥ 0), 37, 38, 41, 42, and 46 exceeded the mean probability by two standard deviations (Fig. 1C). The intervals between “*peak*” index values were non-equidistant. The occurrence of peak NCT values appears to be mainly attributable to increased cumulative duration rather than consecutive duration (Supplementary Figure S5). During BSR≥5, the NCT values diminished below the “*adequate anaesthesia*” index threshold (37) in most cases (Figs. 1E and 2C). The distribution of BSR displayed neither “*peak*” index values nor a specific age distribution pattern (Fig. 2D). Notably, the manufacturer-specified lower threshold for the “*adequate anaesthesia*” range (NCT=37) was the only index value that served as a joint “*peak*” index value for both BSR≥0 (*p* = 0.07) and BSR=0 (*p* = 0.05) conditions. “*Peak*” index values were similarly distributed across all age groups independently of BSR occurrence (Fig. 2 A and B).

### Mean narcotrend values show a significant age-dependent decrease

We found a significant age-dependent decrease in mean NCT index values for both BSR=0 (*p* = 0.038, R^2^ = 0.30, rho=−0.74, Fig. 1B) as well as for BSR≥0 (*p* = 0.003, R^2^ = 0.56, rho=−0.85, Fig. 1D). During BSR≥5 conditions, mean NCT values were persistently in the value range denoting “*deep*” anaesthetic levels, showing no significant variation over age (*p* = 0.79, R^2^ =−0.10, rho=−0.04, Fig. 1 FF). For further description of the linear models, see Supplementary Table 1.

### Adherence of narcotrend values to the manufacturer-recommended index range

**Fig. 3:**
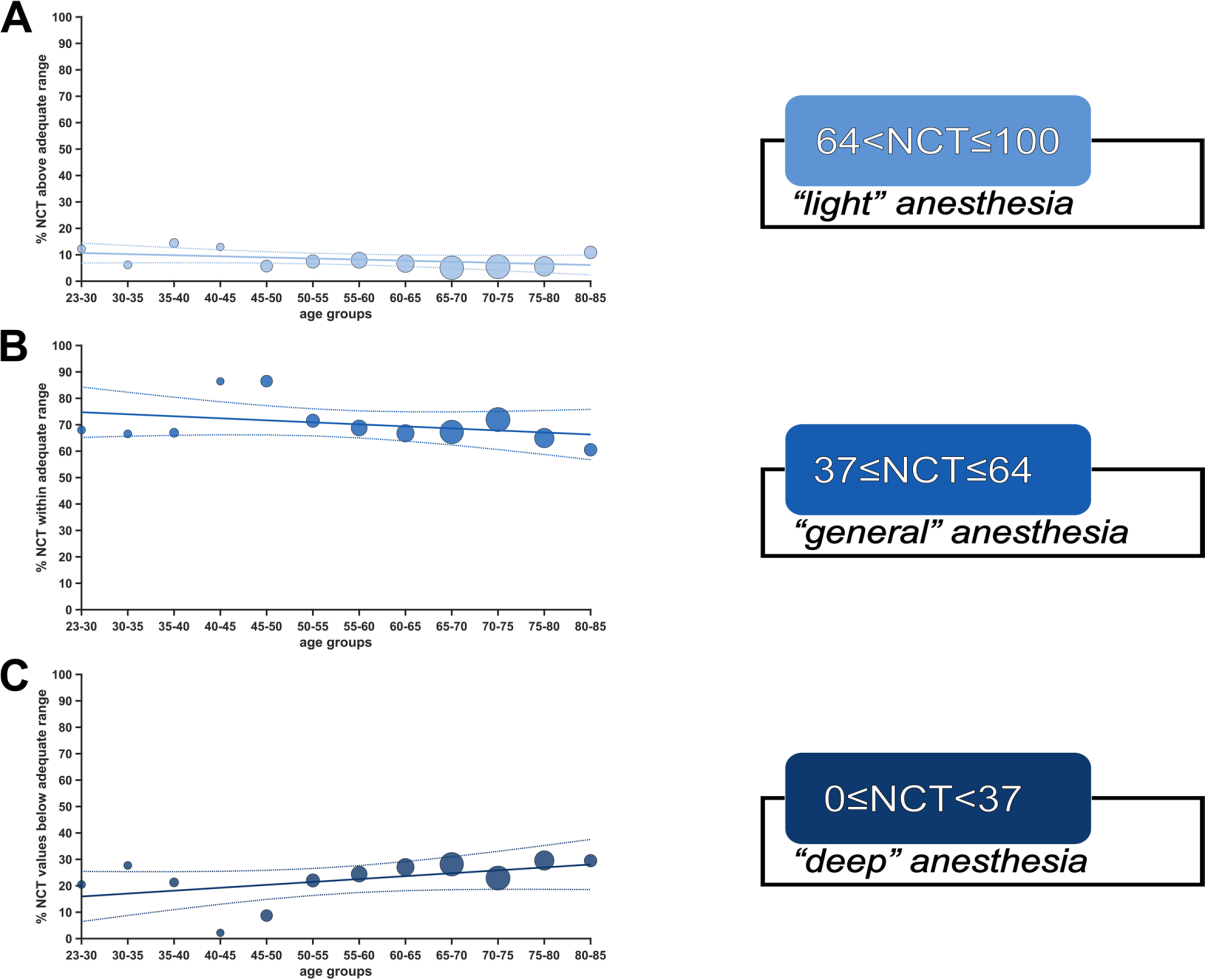
Adherence of Narcotrend (NCT) to the manufacturer-recommended range over age groups during steady-state anaesthesia. Relative distribution of NCT values (BSR=0) in percentages across the NCT index range for age groups. Linear regression fits in the corresponding color and 95th percentile confidence intervals as dotted lines. On the right are the corresponding anaesthetic levels, as described by the manufacturer. **A** Percentages of NCT between 64 and 100, corresponding to an increasing chance of potential wakefulness and recall. **B** Percentages of NCT between 37 and 64, corresponding to “*adequate anaesthesia*” according to the manufacturer. **C** Percentages of NCT between 0 and 37, corresponding to an increasing chance of burst suppression

The NCT showed a high overlap with the recommended range under steady-state anaesthesia conditions (BSR=0), with 70% [Q1,Q3: 67,72] falling within the range, 22% [Q1,Q3: 21,29] below, and 8% [Q1,Q3: 6,12] above (Fig. 3). No significant linear age-related trends were observed. When considering all NCT values (BSR≥0), 60% [Q1,Q3: 55,61] of NCT values fell within the “*adequate anaesthesia*” range, while 33% [Q1,Q3: 27,40] were below and 7% [Q1,Q3: 5,10] above this range. Only for values classified as *“too deep”*, we observed a significant linear increase over age (*p* = 0.018, R^2^ = 0.39, rho = 0.78) (Supplementary Figure S6). Lastly, we examined NCT values with concurrent BSR≥5. For this index constellation, only a small fraction of values (0.09% [Q1,Q3: 0.03,0.16]) were within the “*adequate anaesthesia*” range (portraying contradictory monitor outputs), while 99.91% (Q1,Q3: 99.85,99.96) of NCT values were below range (“*too deep*”), and 0.002% (Q1,Q3: 0,0.003) % above range (“*too light*”) (Supplementary Figure S6). Anaesthesia was maintained with propofol in only 16 cases, but mean NCT values 52 (Q1,Q3: 48,55) did not differ from those observed with volatile anaesthetics 48 (Q1,Q3: 40,57), *p* = 0.968. For the time trend of mean NCT and BSR values with corresponding percentiles across all patients during stable-state anaesthesia, see Supplementary Figure S7.

### Sensitivity and specificity analysis of narcotrend values based on BSR

**Fig. 4:**
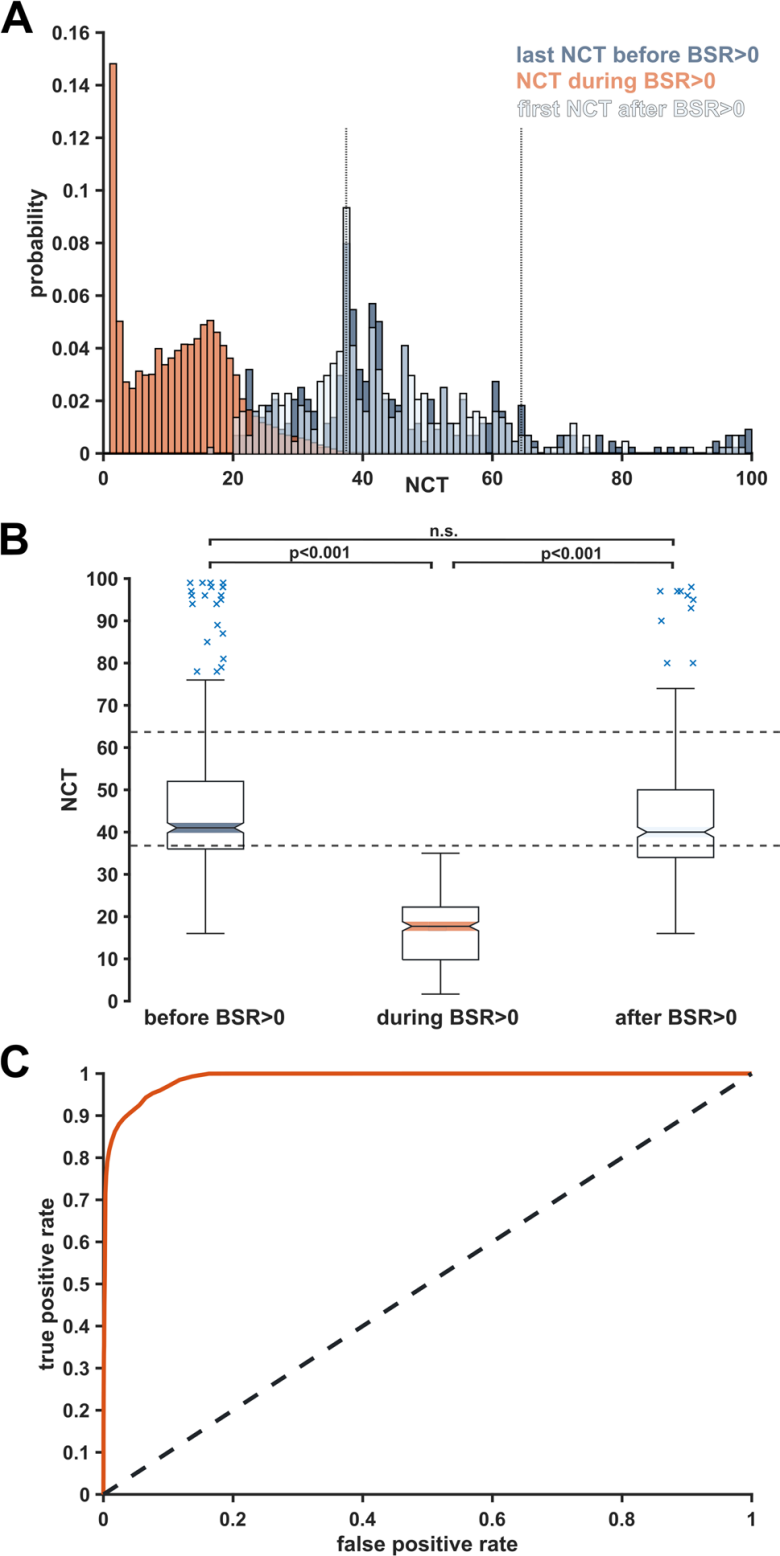
Narcotrend (NCT) value distribution before, during, and after burst suppression ratio > 0 (BSR>0). **A** Histogram showing NCT values recorded immediately before (dark blue), during (red), and immediately after (light blue) BSR > 0 over age groups. Dotted lines indicate the manufacturer’s recommended range. **B** Boxplots showing NCT median, interquartile range (box edges), and 1.5 times the interquartile range (whiskers) for the three conditions. Dotted lines indicate the manufacturer’s recommended range. **C** ROC curve plot for BSR > 0 over NCT index range. The dotted gray line depicts an AUROC of 0.5 (random classifier) and red is the AUROC (0.989) calculated for our data set

We calculated the sensitivity of BSR>0 episodes in predicting NCT values (NCT<37) below the “*adequate anaesthesia*” range, which resulted in a sensitivity of 0.997 [95% CI: 0.996–0.997]. Specificity was calculated by predicting BSR=0 and NCT within the manufacturer-recommended range, resulting in a specificity of 0.695 [95% CI: 0.683–0.705]. We then dichotomised BSR (BSR>0, BSR=0) to compute an AUROC value of 0.989 [95% CI: 0.979–0.992] for the distribution of NCT values (Fig. 4C). BSR>0 triggered an instantaneous decrease in NCT values below the “*adequate anaesthesia*” range, even if the adjacent NCT before and after BSR>0 spread over a broader index range (Fig. 4A). The median NCT immediately before BSR was 41 [Q1,Q3: 36,52]; during BSR>0, it was 12 [Q1,Q3: 4,17]; and immediately after BSR was 40 [Q1,Q3: 34,50]. We found a significant difference (*p* < 0.001) between before/during and during/after. Intriguingly, a non-significant difference (*p* = 0.060) was observed when comparing values recorded immediately before/after BSR (Fig. 4B). Few index values immediately before or after BSR>0 were within the overly *“deep”* index range (0–20), and vice versa. A small fraction (around 0.1%) of NCT values during BSR ≥ 5 was within the “*adequate anaesthesia*” range (Supplementary Figure S6). The cumulative median BSR duration (in minutes [25th, 75th percentiles]) was as follows: 35 [4, 80] for BSR≥5 and 38 [5, 86] for BSR>0. The corresponding consecutive median duration (in seconds [25th, 75th percentiles]) was 38 [22, 80] for BSR ≥ 5 and 36 [26, 47] for BSR> 0.

### Non-interpretable monitor outputs of the narcotrend

**Fig. 5:**
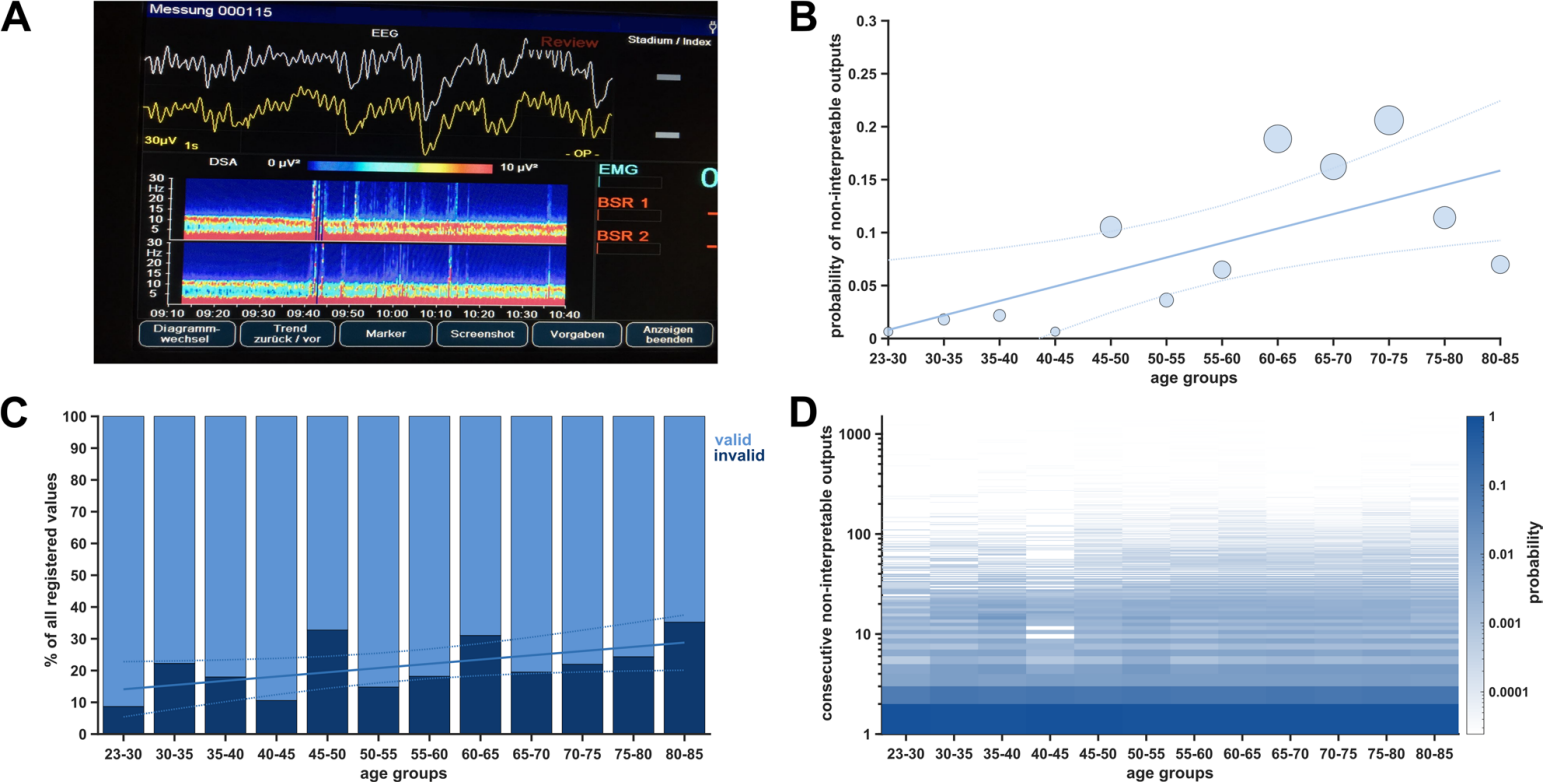
Occurrence of non-interpretable monitor outputs over age groups. **A** Narcotrend monitor displaying *“- -*“ when it cannot present an index value. **B** Scatter plot showing absolute probability (normalized over all age groups to 1) for non-interpretable outputs. Linear regression fit in the corresponding color and 95th confidence intervals as dotted lines. The size of the dots was scaled to the number of patients available per age bin. **C** Stacked histogram showing percentiles of non-interpretable outputs relative to all registered outputs (normalized over each age group to 100). Linear regression fit in the corresponding color and 95th confidence intervals as dotted lines. **D** Heat map showing consecutive runtime of non-interpretable outputs in seconds over age groups

The NCT Monitor is expected to continuously present numeric values (0–100). If the monitor cannot do so, it displays the symbol: *“- -”*(Fig. 5A). We found heightened probabilities and percentages of non-interpretable monitor outputs among elderly patients. This is pertinent for both the overall probability (*p* = 0.013, R^2^ = 0.42, rho = 0.79, Fig. 5B) as well as for the percentage relative to each patient’s total NCT values (*p* = 0.050, R^2^ = 0.26, rho = 0.59, Fig. 5 C). We calculated the median cumulative percentage of surgery time with non-interpretable monitor output as 12% [Q1,Q3: 6,15]. Age, however, did not significantly influence the consecutive duration of non-interpretable values (Fig. 5D). The consecutive duration remained consistently low, with a median of 1 s. Given the high frequency of non-interpretable outputs, we analysed the first NCT value after each non-interpretable output. We matched the distribution of these values with the original “*baseline*” distribution of NCT (Supplementary Figure S8). They closely mirrored the original probability distribution. Subtracting NCT values immediately adjacent to non-interpretable output from one another resulted in zero for most cases. The frequent occurrence of non-interpretable outputs does not significantly affect the overall distribution of index values. While non-interpretable NCT values can occur during concurrent burst suppression (BSR>0), the majority (probability > 0.9) were observed in the absence of simultaneous burst suppression (see Supplementary Figure S8). Thus, burst suppression does not appear to be the primary cause of non-interpretable monitor outputs. Across the full NCT range, the probability of a given value being immediately preceded or followed by a non-interpretable monitor output was largely uniform, with higher probabilities observed for NCT values between 20 and 30 and > 80 (Supplementary Fig. S9).

## Discussion

In this retrospective analysis of patients undergoing cardiac surgery, we evaluated the performance of the Narcotrend monitor. While most values during the maintenance of anaesthesia were within the manufacturer-recommended range, we also found conspicuous “*peak*” index values with a higher probability of occurrence. Further, while the onset of burst suppression triggered a near-instant decrease in NCT values, NCT values recorded immediately before and after BSR>0 demonstrated considerable variability. Lastly, while non-interpretable monitor outputs significantly increased with age, as reported for another data set [15], there was no significant difference across age groups in the consecutive duration of these non-interpretable values.

### Comparability between commercial “*depth*” of anaesthesia monitoring devices

Neuromonitoring indices are designed to help anaesthesiologists in adjusting anaesthetic dosing levels. Although medical societies recommend their use [1], widespread uncertainty impedes their routine application and integration into clinical practice [16]. Although this study did not include other EEG-based depth of anaesthesia monitors, differences in index scaling between devices are well documented. For example, studies comparing BIS and NCT under propofol–remifentanil anaesthesia found only ~ 50% agreement in their respective “*adequate*” ranges, with substantial proportions classified as deeper or lighter anaesthesia by one device compared to the other [17]. Such variability illustrates that classification into manufacturer-defined ranges is monitor-specific, which should be considered when interpreting our findings. The NCT algorithm additionally incorporates patient age into its classification, but the details of this adjustment are not disclosed by the manufacturer. In a study that compared five different indices [15], only the NCT showed no substantial increase with patient age, consistent with results seen in an earlier analysis of this data set [12].

### Occurrence of “*peak index*” values

Certain “*peak*” NCT index values occurred with disproportionately high frequency, exceeding the mean probability by more than two standard deviations. We recently observed a similar phenomenon with the GE Entropy™ module, where State Entropy values also exhibited conspicuous clustering [18]. The clinical implications of these distributional patterns remain unclear. Of particular note is the frequent occurrence of NCT=37, the most common peak in our dataset, which marks the lower threshold of adequate anaesthesia. Our findings suggest that the scaling behaviour of the index at EEG patterns associated with deeper anaesthesia may be more stationary. This raises the question of whether dynamic titration of anaesthetics at these stages is reliably reflected by the monitor. The occurrence of “*peak*” values may be influenced by various factors, such as age-related EEG changes, artefacts or EMG interference, computational latency, and patient-specific variability, but this remains speculative and may not be attributed solely to anesthetic management.

### Non-interpretable narcotrend monitor outputs and BSR

Our findings add to the existing literature that the NCT tends to display non-interpretable outputs [15]. We now provide a more detailed characterisation of this behaviour, demonstrating that these brief interruptions occur predominantly when no burst suppression is present (BSR=0), indicating that burst suppression is unlikely to be the primary cause of non-interpretable outputs. Specifically, the NCT monitor experienced data calculation issues 7% of the time during anaesthesia maintenance, whereas the BIS monitor exhibited these problems only 3% of the time [19]. The NCT also proved a higher sensitivity to suspected artefacts (12.6 ± 1.0%) than the BIS (0.4 ± 0.1%) algorithm. BIS’s performance in predicting propofol effect-site concentrations remained consistent regardless of whether data flagged as artefacts by the NCT was included or excluded [20]. A study examining loss and return of consciousness found that the interval between valid indices ranges from 3 s to multiple minutes [21]. Given our finding that the median percentage of surgery time with non-interpretable monitor outputs was substantial (12% [Q1,Q3: 6,15]), but the duration of consecutive non-interpretable outputs was brief (median of 1 s), we recommend a pragmatic approach of retaining the last valid NCT index value – at least in less dynamic clinical situations. A potential limitation to the performance of the Narcotrend is the age-related increase in non-interpretable monitor outputs, particularly during anaesthesia management in elderly patients. In this context, detecting burst suppression should help as it constitutes an objective indicator of overly “*deep*” anaesthesia and potentially predicts postoperative delirium in cardiac surgery patients [22]. However, discrepancies exist between monitors due to algorithm variations and the underestimation of burst suppression by automated BSR systems compared to visual examination [23]. Additionally, both the GE Entropy™ and BIS indices can contradictorily display index values of “*adequate anaesthesia*” despite BSR>0 [14, 24]. Our results show that the NCT intrinsically precludes contradicting index constellations, as BSR>0 always concurs with index values below the “*adequate anaesthesia*” range. We hypothesize an underlying technical characteristic to interconnect the NCT algorithm and BSR (in line with previously described approaches to burst suppression correction, e.g., the burst-compensated spectral edge frequency [25]), as the monitor displays a broad range of index values immediately before and after BSR>0. The observed heterogeneity in pre- and post-burst suppression indices may be attributed to a real heterogeneity in EEG characteristics and index calculation time delays [26]. Predicting burst suppression based on EEG features and integrating such predictions into commercial monitors remains a task for the future. A recent study, for example, employed state-space methods to track transitions from slow–delta–alpha oscillations to burst suppression and identified two dynamical processes (alpha-wave amplitude and slow-wave frequency modulation) that continuously modulate the intermediate dynamics between these states. This approach may provide the foundation for a future predictive model [27].

### Narcotrend and the adequacy of anaesthesia

In a large cohort with over 4,000 patients, 7.3% of patients showed anaesthesia classified as too “*light*”, while 16.8% showed very “*deep*” anaesthesia (NCT: 0–12) [28]. Our study confirms these findings and adds that for BSR=0 conditions, 70% [Q1,Q3: 67,72] fall within the range, 22% [Q1,Q3: 21,29] below, and 8% [Q1,Q3: 6,12] above the range. Another study found that specific NCT values occur more frequently than others, with the index remaining unchanged for several minutes, effectively “*resting*” on these values [29]. We described a similar phenomenon for the GE Entropy™ module indices [18] and can now confirm this for NCT index values (Fig. 1). However, the occurrence of “*peak*” values in this study was primarily attributable to increased cumulative duration compared to consecutive duration (see Supplementary Figure S5). While employing NCT to guide anaesthetic “*depth*” led to a significant decrease in vasopressor dosage compared to the conventional clinical approach (despite reporting NCT values below the “*adequate anaesthesia*” range), adverse events like intraoperative movement or irregular breathing still occurred in another study [30]. Lastly, the comparability of the “*adequate anaesthesia*” index ranges in five different processed EEG indices showed that when analysing identical raw EEG signals, these monitors agreed in only one-third of cases and disagreed in two-thirds. In approximately half of the cases, the monitors correctly identified “*light*” hypnotic depth, while one-third suggested excessive “*depth*” [10]. As long as manufacturers do not fully disclose whether their recommended ranges for supposedly “*adequate anaesthesia*” are linked to verifiable clinical endpoints, they should be used cautiously in clinical settings.

### Limitations

Given the retrospective nature of our study, all associations described in this study are descriptive. Several parameters were either omitted or unavailable for analysis. We did not factor in drug dosages and other parameters such as heart rate, blood pressure, and ventilation settings. Nonetheless, the Narcotrend index does not account for most of these parameters, except for patient age. This study was restricted to patients undergoing cardiac surgery with CPB. Although processed EEG indices do not directly account for the type of intervention, our findings formally apply only to this population. Further, the cardiac surgery context of our data may lead to a higher incidence of BSR than expected in elective surgery. This study also did not include independent measures of anaesthetic depth to validate the NCT classification. Therefore, the term “*adequate anaesthesia*” refers strictly to the manufacturer-defined index range. The age-related decline in NCT values may reflect either physiological EEG changes or algorithmic processing effects. Current evidence, however, tends to support a physiological explanation [15]. The observation that a broad range of NCT values was recorded immediately before and after BSR >0 may partly be attributable to the time delay in NCT index calculation.

### Conclusion

Our analysis of a predominantly male patient cohort undergoing cardiac surgery reveals that during steady-state anaesthesia, most index values fall within the manufacturer-recommended range for adequate anaesthetic “*depth*”. The Narcotrend showed an age-dependent decrease in its mean index values. At the same time, we found an age-dependent increase in absolute and relative durations of non-interpretable outputs. These constellations only lasted briefly, but their high incidence during “*steady-state*” without simultaneous burst suppression detection (BSR=0) could lead to uncertainties in index interpretation in elderly patients. BSR>0 displays near-perfect discriminatory ability in predicting NCT values below the “*adequate anaesthesia*” range in all age groups. This points towards a technical interconnection between the NCT and BSR algorithms. The underlying matching of the raw EEG to processed NCT seems conspicuous, as we found “*peak*” index values with a higher probability of occurrence than others, challenging the continuous scaling of NCT index values.

## Supplementary Information

Supplementary Material 1.

## Data Availability

The dataset generated and analysed during the current study is available from the corresponding author on reasonable request.

## Abbreviations

AUC: Area Under the Curve
BSR: Burst Suppression Ratio
EEG: Electroencephalogram
NCT: Narcotrend
ROC: Receiver Operating Characteristic
