# Supplementary material for: Evaluating the performance of the NarcotrendR EEG index during anaesthesia for cardiothoracic surgery: a single-centre retrospective study
Source: BMC Anesthesiol. 2025 Nov 14;25:559. doi: 10.1186/s12871-025-03500-5 (PMC12619384; doi:10.1186/s12871-025-03500-5)
Supplement: Supplementary file 1 — Supplementary Material 1. [file 12871_2025_3500_MOESM1_ESM.pdf]

Supplementary material to

Evaluating the Performance of the Narcotrend[R] EEG Index during Anaesthesia for Cardiothoracic Surgery: A Single-Centre Retrospective Study

Authors:

Max Ebensperger, Matthias Kreuzer, Stephan Kratzer, Darren Hight, Heiko A. Kaiser, Gerhard Schneider, Stefan Schwerin.

Corresponding author: Stefan Schwerin, stefan.schwerin@tum.de,

Department of Anesthesiology and Intensive Care, School of Medicine and Health, Technical University of Munich, Munich, Germany

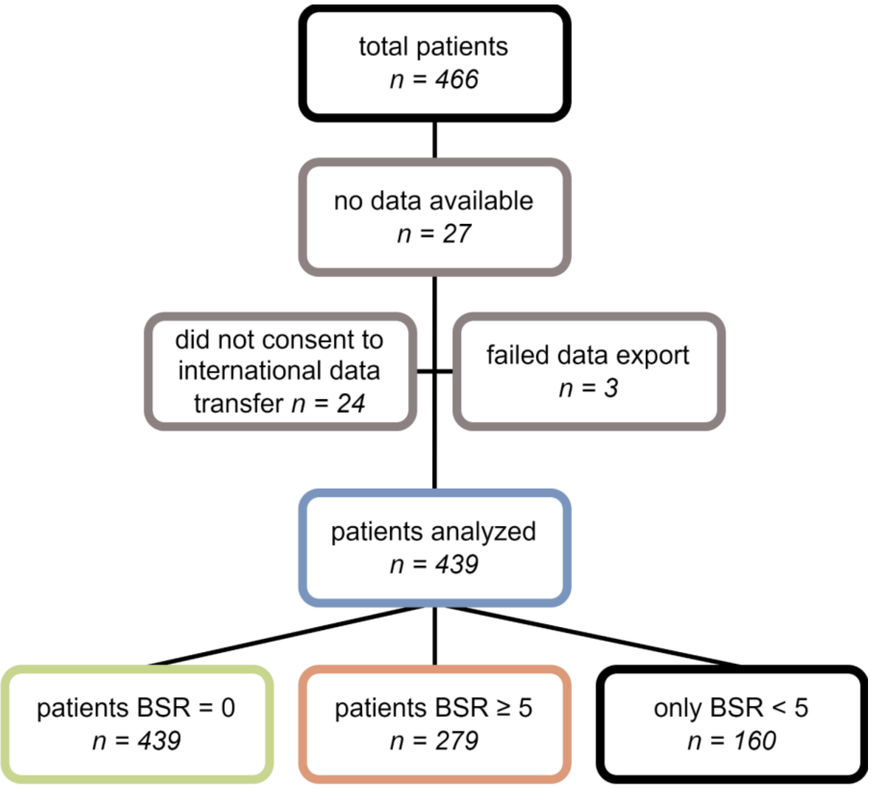

Fig. S1 Flowchart illustrating patient groups as well as exclusion criteria.

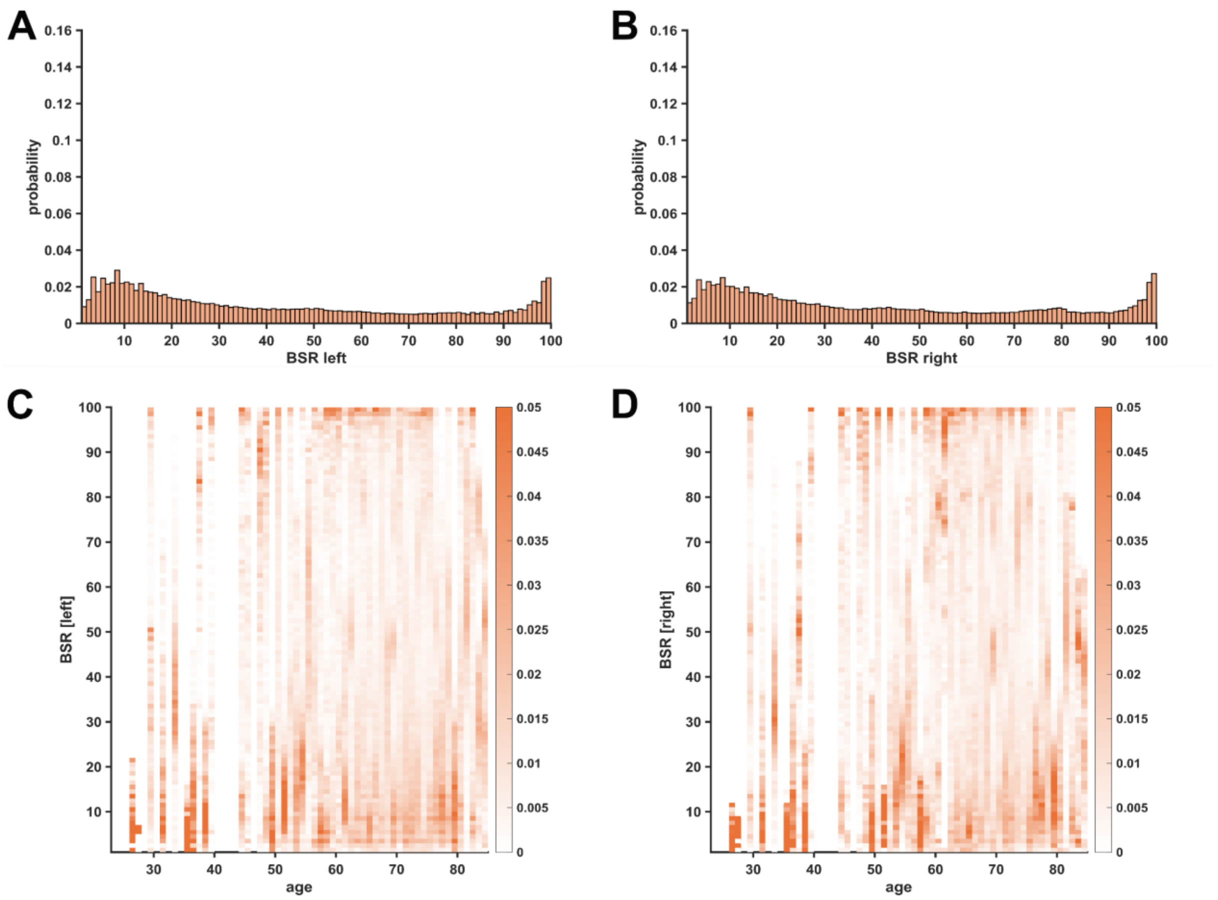

Fig. S2 Histograms and Heat maps for left and right burst suppression ratio (BSR)

A) Histogram of the left BSR index value probability

B) Histogram of the right BSR index value probability

C) Heat map of left BSR left over all ages

D) Heat maps of BSR right over all ages

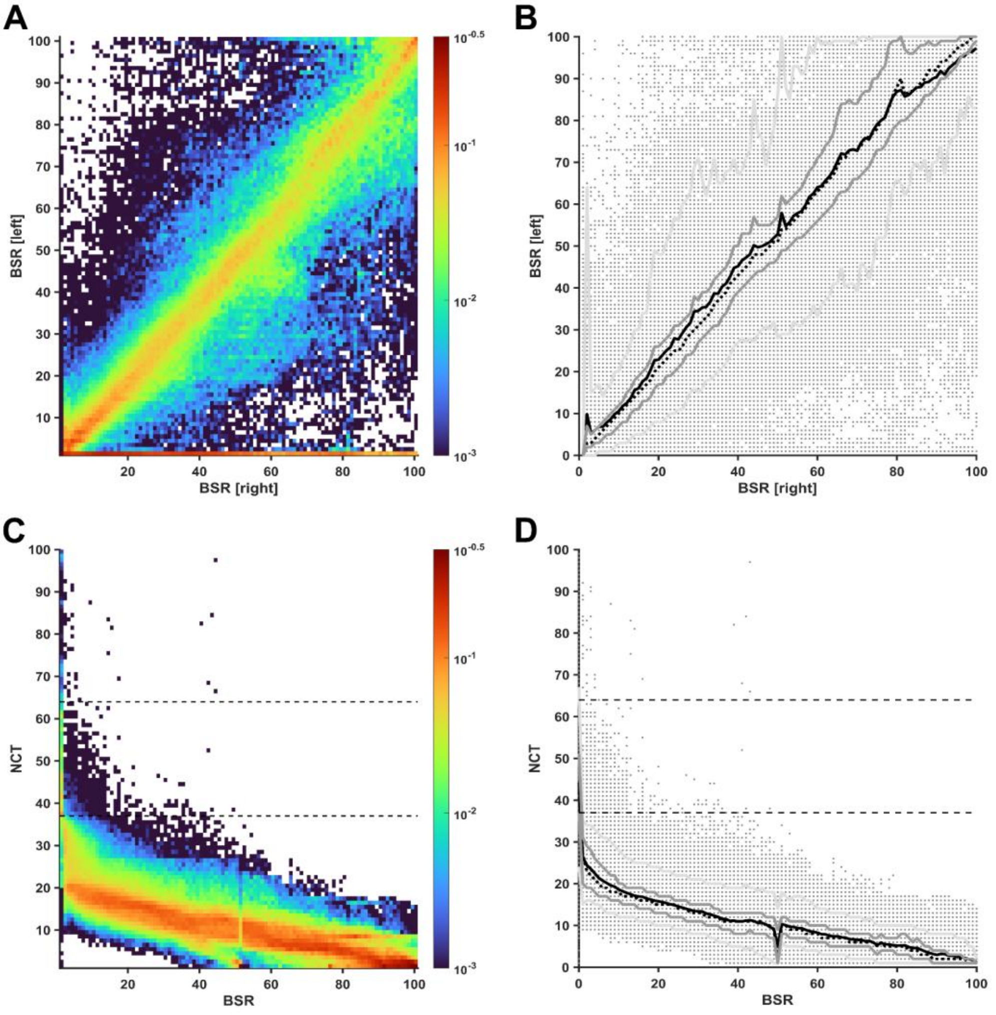

Fig. S3 Heat maps showing overlap between simultaneously recorded burst suppression ratio (BSR) values by left and right electrodes, as well as overlap between simultaneously recorded Narcotrend (NCT) and BSR values.

A) Heat map showing BSR (left) over BSR (right) with the normalized probability as color bar.

B) Heat map for BSR (left) over BSR (right) with median (black), mean (dotted) and 95th, 5th,75th and 25th percentiles (grayscale)

C) Heat map showing NCT over BSR with the normalized probability as color bar.

D) Heat map for NCT over BSR (mean) with median (black), mean (dotted) and 95th, 5th, 75th and 25th percentiles (grayscale)

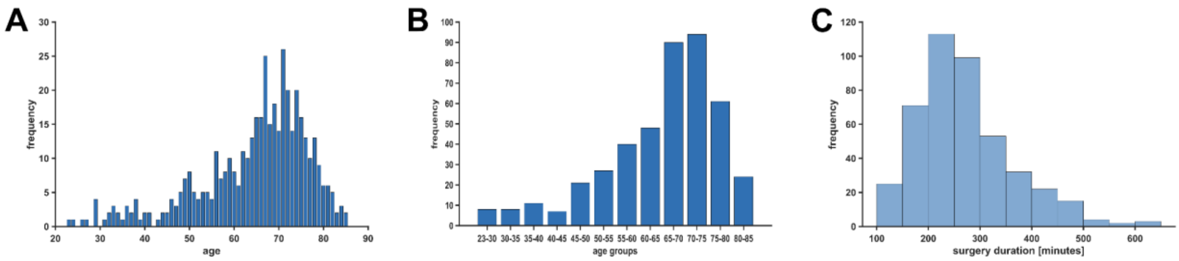

Fig. S4 Frequency distribution of patient age and surgery duration

A) Histogram of patient age with 1-year bins.

B) Histogram of patient age with 5-year bins.

C) Histogram of surgery duration (minutes).

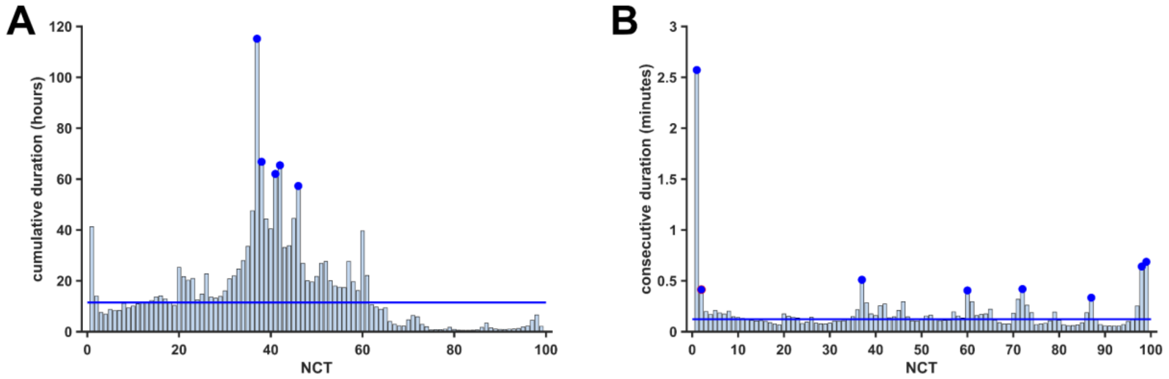

Fig. S5 Cumulative and consecutive durations of individual NCT index values

"*Peak*" NCT index values (marked with blue dots) were identified as exceeding a duration of twice the standard deviation (indicated with a horizontal blue line) for overall durations.

A) Cumulative durations in hours. "*Peak*" NCT index values included: 37, 38, 41, 42, and 46.

B) Consecutive durations in minutes. *"Peak"* NCT index values included*:* 1, 2, 37, 60, 72, 87, 98, and 99.

Supplementary Table 1 of results for linear regression models with corresponding Spearman's correlation coefficients.

All linear models describe the relationships of NCT index value constellations/combinations listed in the first column and patient age. Abbreviations: %, percentage; BSR, burst suppression ratio; adj., adjusted; CI, confidence interval; non-interp., non-interpretable.

	**linear model**	**CI of slope**	**adj. R^2**	**P-value**	**Spearman´s rho**
**% BSR≥0 above**	-0.49*age+10.42	(-1.01, 0.03)	0.24	0.062	-0.50 (-0.88, 0.16)
**% BSR≥0 within**	-1.61*age+70.16	(-3.23, 0.01)	0.26	0.051	-0.73 (-0.93, -0.25)
**% BSR≥0 below**	2.10*age+19.41	(0.44, 3.76)	0.39	0.018	0.78 (0.38, 0.93)
**% BSR=0 above**	-0.41*age+11.06	(-0.99, 0.17)	0.12	0.144	-0.50 (-0.99, 0.17)
**% BSR=0 within**	-0.77*age+75.51	(-2.24, 0.70)	0.03	0.271	-0.31 (-0.85, 0.44)
**% BSR=0 below**	1.10*age+14.85	(-0.36, 2.56)	0.14	0.124	0.68 (0.10, 0.96)
**% BSR≥5 above**	0.0002*age+0.0006	(-0.0004, 0.0008)	-0.04	0.462	0.53 (-0.05, 0.85)
**% BSR≥5 within**	0.02*age-0.02	(0.008, 0.03)	0.57	0.003	0.79 (0.36, 0.95)
**% BSR≥5 below**	-0.01*age+100,01	(-0.024, -0.004)	0.48	0.007	-0.77 (-0.96, -0.30)
**mean BSR≥0**	-0.96*age+47.08	(-1.52, -0.41)	0.56	0.003	-0.85 (-1, -0.4)
**mean BSR=0**	-0.54*age+49.16	(-1.04, -0.04)	0.30	0.038	-0.74 (-0.97, -0.26)
**mean BSR≥5**	+0.03*age+10.24	(-0.21, 0.27)	-0.10	0.79	-0.04(-0.63, 0.63)
**Probability non-interp.**	0.014*age-0.006	(0.004, 0.96)	0.42	0.013	0.79 (0.31, 0.96)
**% non-interp.**	1.34*age+12.78	(-0.003, 2.67)	0.26	0.050	0.59 (0.03, 0.96)
**time non-interp.**	0.79*age+6.03	(-0.05, 1.63)	0.24	0.063	0.51 (-0.19, 0.92)

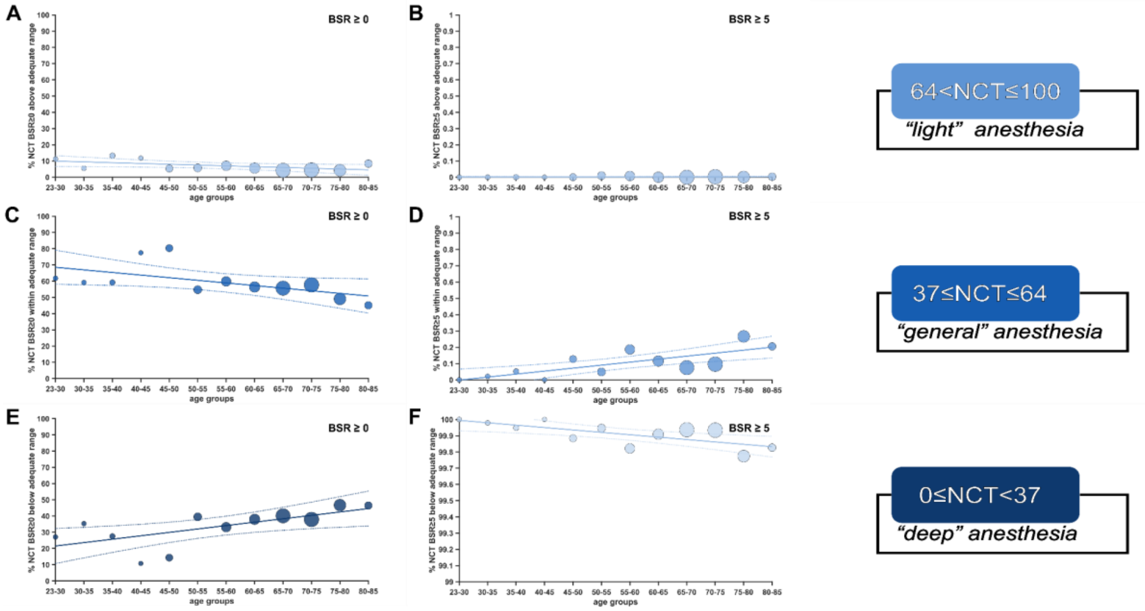

Fig. S6 Percentage distribution of Narcotrend (NCT) values with corresponding BSR≥0 and BSR≥5 in relation to the manufacturer-recommended range

A) Percentage of NCT values between 64–100 during anesthesia for BSR≥0, corresponding to an increasing chance of potential wakefulness and recall.

B) Percentage of NCT values between 64–100 during anesthesia for BSR≥5.

C) Percentage of NCT values between 37–64 during anesthesia for BSR≥0, corresponding to an adequate anesthetic range.

D) Percentage of NCT values between 37–64 during anesthesia for BSR≥5.

E) Percentage of NCT values between 0–37 during anesthesia for BSR≥0, corresponding to an increasing chance of burst suppression.

F) Percentage of NCT values between 0–37 during anesthesia for BSR≥5.

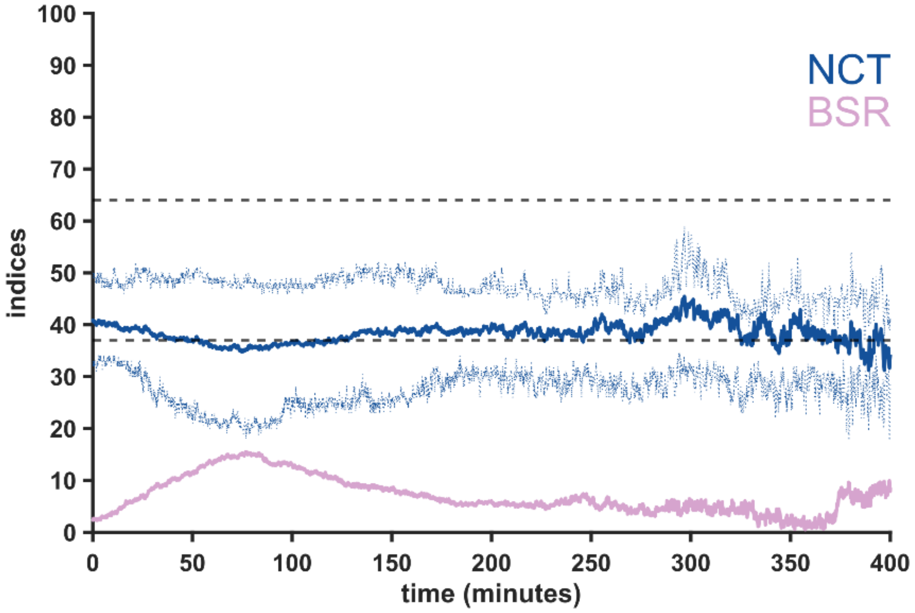

Fig. S7 Mean NCT and BSR values of all patients during steady-state anaesthesia

The x-axis represents the timeline across all patients, excluding the initial and final 20 minutes of data recording. The y-axis displays the NCT values, with the mean (dark blue) and the 25th and 75th percentiles (light blue), as well as mean BSR (pink). Dashed lines mark the upper and lower threshold for “adequate anesthesia” by the manufacturer (NCT=37 and NCT=64).

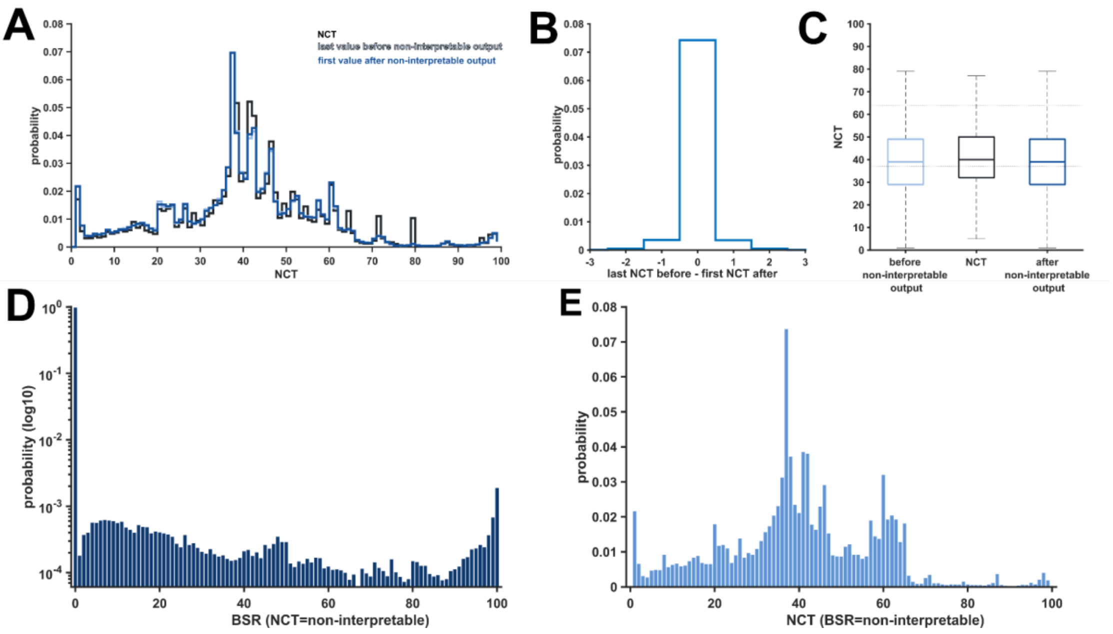

Fig. S8 Comparison of NCT values immediately before and after non-interpretable outputs and consecutive occurrence of non-interpretable outputs

A) Superimposed stair plots with the overall probabilities (normalized to 1) of valid NCT values (light blue) and first valid NCT values after non-interpretable output occurrences (dark blue). Overall non-interpretable output probability in black. Non-interpretable monitor outputs do not substantially impact overall NCT value distributions. *"Peak"* index values remain discernible.

B) Cumulative mean difference between the last NCT value before non-interpretable outputs, minus the first valid NCT value after non-interpretable outputs.

C) Boxplots showing the mean NCT before, after, and excluding non-interpretable values.

D) Histogram with the BSR index distribution with concurrently recorded non-interpretable NCT values. BSR=0 shows the largest probability, with >0.9 overall probability (probability in log scale).

E) Histogram with the NCT index distribution with concurrently recorded non-interpretable BSR values. This resembles the overall NCT index distribution.

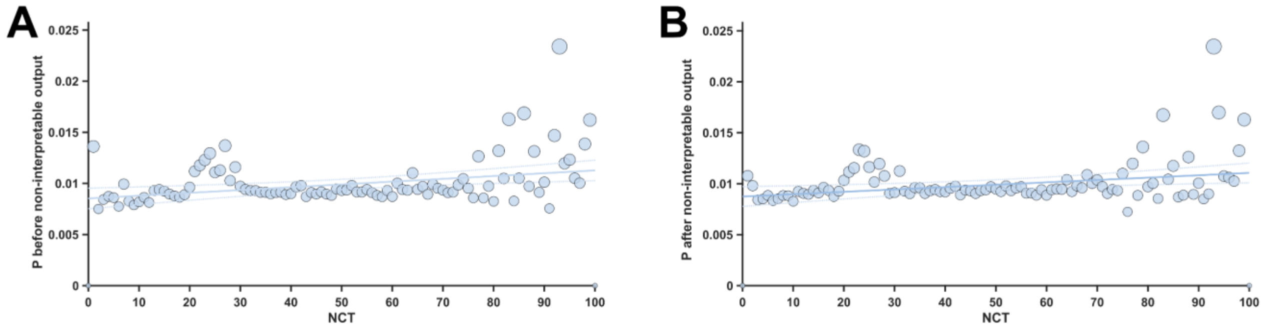

Fig. S9 Probability of a non-interpretable monitor output in relation to corresponding NCT index values.

A) Probability of a non-interpretable monitor output occurring *before* respective NCT index values

B) Probability of a non-interpretable monitor output occurring *after* respective NCT index values
